# Organ-on-a-Chip for Studying Gut-Brain Interaction Mediated by Extracellular Vesicles in the Gut Microenvironment

**DOI:** 10.3390/ijms222413513

**Published:** 2021-12-16

**Authors:** Min-Hyeok Kim, Danny van Noort, Jong Hwan Sung, Sungsu Park

**Affiliations:** 1 School of Mechanical Engineering, Sungkyunkwan University (SKKU), Suwon 16419, Korea; mhkim967@g.skku.edu; 2 Division of Biotechnology, Linköping University, 58183 Linköping, Sweden; drr.dvn@gmail.com; 3 Centro de Investigación en Bioingeniería, Universidad de Ingenieria y Tecnologia—UTEC, Lima 15063, Peru; 4 Department of Chemical Engineering, Hongik University, Seoul 04066, Korea; 5 Department of Biophysics, Institute of Quantum Biophysics (IQB), Sungkyunkwan University (SKKU), Suwon 16419, Korea

**Keywords:** extracellular vesicles, gut-brain axis, multi-organ-on-a-chip, exosomes, blood-brain barrier, pathophysiology

## Abstract

Extracellular vesicles (EVs) are a group of membrane vesicles that play important roles in cell-to-cell and interspecies/interkingdom communications by modulating the pathophysiological conditions of recipient cells. Recent evidence has implied their potential roles in the gut–brain axis (GBA), which is a complex bidirectional communication system between the gut environment and brain pathophysiology. Despite the evidence, the roles of EVs in the gut microenvironment in the GBA are less highlighted. Moreover, there are critical challenges in the current GBA models and analyzing techniques for EVs, which may hinder the research. Currently, advances in organ-on-a-chip (OOC) technologies have provided a promising solution. Here, we review the potential effects of EVs occurring in the gut environment on brain physiology and behavior and discuss how to apply OOCs to research the GBA mediated by EVs in the gut microenvironment.

## 1. Introduction

Extracellular vehicles (EVs) are a group of phospholipid bilayer vesicles that originate from diverse types of cells, from prokaryotic cells to eukaryotic cells [1,2]. In the past, EVs were considered a type of membrane debris [3,4]. Since then, accumulating evidence has challenged this idea and revealed that EVs play a vital role in intercellular communication [5,6]. In the human body, EVs can be observed in almost all types of body fluids, such as blood, urine, saliva, cerebrospinal fluid, bronchoalveolar lavage fluid, amniotic fluid, seminal plasma, and breast milk [7,8]. They play important roles in the homeostasis of the body and disease processes by participating in intercellular signaling [9]. For example, EVs produced by both immune and non-immune cells regulate body immunity [10], and EVs could be involved in neurodegenerative diseases such as Alzheimer’s disease (AD) and Parkinson’s disease (PD) [11].

**Figure 1:**
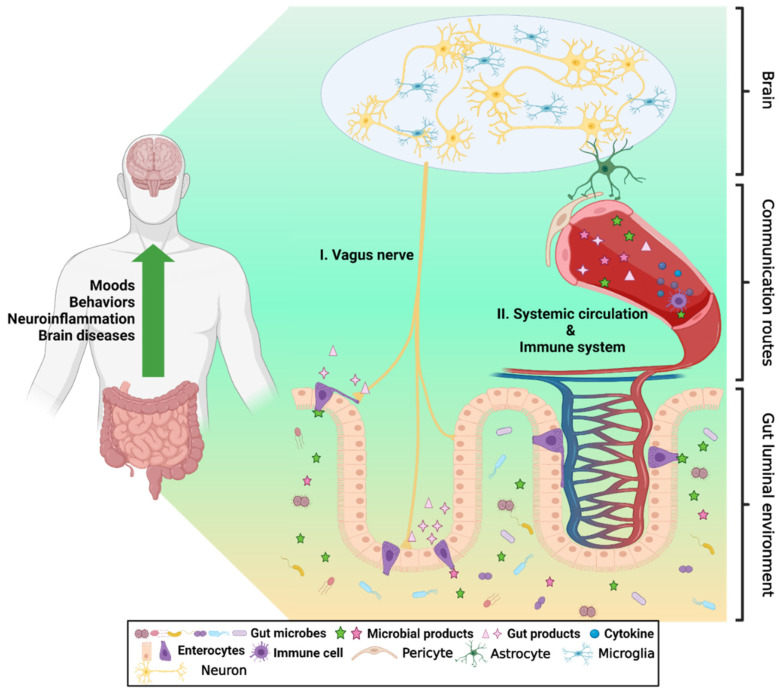
**Gut-brain axis.** The gut luminal environment, including the gut microbiome, affects the physiology and behavior of the brain via multiple routes. (**I.**) The vagus nerve senses gut hormones and microbial metabolites from the gut environment and delivers the signals to the brain. (**II.**) Microbial products can cross the gut epithelial barrier, which makes them eventually enter systemic circulation. Additionally, some gut hormones are secreted into the bloodstream, stimulating the immune system or traveling to the BBB, which affects changes in the physiology and behavior of the blood-brain barrier (BBB) and brain. This illustration was created on BioRender.com.

It has been revealed that complex bidirectional interactions exist between the gut microenvironment and the brain, which is called the gut-brain axis (GBA) [12,13]. Alterations in gut physiology or environment by diet patterns or microorganisms may contribute to physiological changes in the brain and vice versa. GBA contains multiple signaling pathways, and these pathways through the vagus nerve and blood circulation have been well studied. The vagus nerve senses gut hormones and microbial metabolites and delivers the signals to the central nervous system (CNS) (Figure 1) [14,15]. In addition, microbial products can cross the gut epithelial barrier, which allows them to enter systemic circulation and eventually alter brain physiology and behavior (Figure 1) [16]. It has been recently understood that microbial EVs are key mediators of these interactions [14,17]. Several recent reports imply that gut-released EVs could be involved in this communication.

In the gut microenvironment, EVs can originate from gut cells and microorganisms. However, the roles of these EVs in GBA have been less highlighted. In addition, the current models of GBA and analyzing techniques for EVs have many limitations. For example, animal models mainly used in GBA research [18] show poor experimental reproducibility [19], and traditional in vitro well-plate models lack physiological relevance to the in vivo human body due to the absence of a dynamic environment and perfusion [20]. In addition, the practical use of EVs has been limited due to critical bottlenecks, such as lack of standardized protocols and extensive labor during sample preparation [21,22,23,24].

Recent advances in organ-on-a-chip (OOC) technologies can potentially substitute current GBA models. OOCs aim to recapitulate the physiological environment and functionality of human organs on a chip by mimicking the crucial organotypic cellular architecture and functionality, 3D extracellular matrix, biochemical factors, and biophysical cues [25,26]. Recently, a few GBAs-on-a-chip have been developed, while several studies have simulated EV-mediated disease models using OOCs. Therefore, OOCs can have great potential for studying the GBA mediated by EVs in the gut microenvironment.

The purpose of this review is to highlight the importance of EVs in the context of the GBA and discuss how to utilize OOCs in the research on GBA communication mediated by EVs. For this purpose, we collected recent studies on EVs in the gut microenvironment, categorizing them into several subgroups based on their origins. Additionally, we reviewed their potential effects on the pathophysiology of the brain. Here, we discuss the design principles of GBA-on-a-chip for EV research and review several other EV-mediated disease models using OOCs. We hope that this review will provide new insights into understanding complex GBA systems.

## 2. Involvement of Gut-Originated EVs in the GBA

### 2.1. Type and Functions of Gut-Originated EVs

EVs in the gut microenvironment can be categorized into two groups based on their origin: microorganisms and host cells.

**Figure 2:**
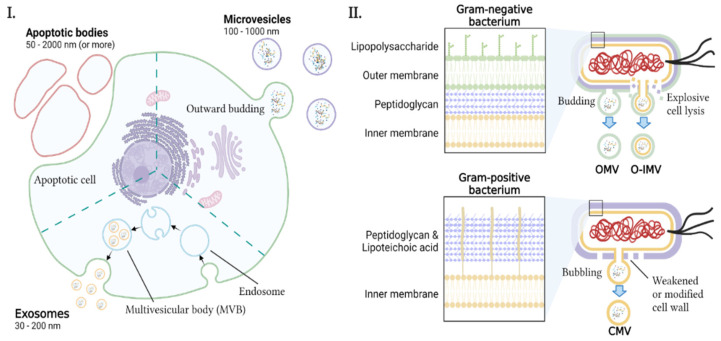
Biogenesis of different EVs. Eukaryotic cells and bacteria produce distinct kinds of EVs. (**I.**) Eukaryotic cells secrete various EVs such as exosomes, microvesicles, and apoptotic bodies. Exosomes are generated through inward budding in late endosomes in the cytoplasm, carried by multivesicular bodies, and eventually released into the extracellular environment. Microvesicles are directly generated from the plasma membrane via outward budding. Apoptotic bodies are released from dying cells. (**II.**) Both Gram-negative and Gram-positive bacteria can produce bacterial membrane vesicles. However, among them, outer membrane vesicles (OMVs) from Gram-negative bacteria are more common than outer-inner membrane vesicles (O-IMVs) or cytoplasmic membrane vesicles (CMVs). OMVs are simply budded from the outer membrane of Gram-negative bacteria, while the inner membrane and cell wall are stable during this process. Some species of Gram-negative bacteria also produce O-IMVs whose formation is followed by explosive cell lysis of Gram-negative bacterium through phage-induced endolysin-triggered cell wall breakdown, which leads cell death. As their name suggests, O-IMVs feature both outer and inner membranes originating from the parent cell, while OMVs and CMVs house only one membrane. CMVs can be observed from Gram-positive bacteria. However, the exact biogenesis and release mechanism is still unclear. One of the leading theories is generation and release after cell wall modification by phage-induced endolysin. This not only causes CMV formation but also leads “bubbling cell death”. This illustration was created on BioRender.com.

EVs from eukaryotic cells and bacteria are summarized in Figure 2 and Table 1. Bacterial EVs (BEVs) are crucial mediators of microbiota–host interactions [27]. Most EVs produced by Gram-negative bacteria originate from the outer membrane through budding. However, some EVs containing both the outer and inner membranes of Gram-negative bacteria have been recently reported in some species [28,29,30]. The former is called outer membrane vesicles (OMVs), and the latter is outer-inner membrane vesicles (O-IMVs). Recent studies have demonstrated that EVs can be produced from Gram-positive bacteria and are related to diseases such as *Staphylococcus aureus*-induced atopic dermatitis [31]. Gram-positive bacteria produce cytoplasmic membrane vesicles (CMVs), which are thought to be released by the incorporation of cell wall modifications [14,31,32,33]. However, the exact release mechanisms of CMVs are still not clearly understood [32,33]. Protozoans are also important eukaryotic microorganisms in the gut microenvironment. Their EVs have also been reported to be involved in host pathology [34,35,36]. In addition to bacteria and protozoans, the human gut can harbor many other microorganisms, such as fungi, whose EVs can also interact with host cells and participate in pathogenesis [37,38,39].

There is little consensus on the classification of EVs in eukaryotes, but they can be categorized into several subtypes, including microvesicles (MVs), exosomes, and apoptotic bodies (ABs), which are based on their size, release pathway, biogenesis, content, and function [28,42,49]. MVs are formed directly from the plasma membrane via outward budding [42]. Their sizes typically range from 100 nm to 1 μm in diameter [42]. Exosomes are constitutively generated from late endosomes, formed by inward budding of the limited multivesicular body membrane [40]. The size of exosomes can range from 30 to 200 nm, but this range varies depending on the authors [42,50]. ABs are released by dying cells into the extracellular space; they are reported to range in size from 50 to 2000 nm or more [42,49]. Under specific conditions, they are more abundant than MVs or exosomes [49].

EVs contain lipids, proteins, and various nucleic acid species from the parent cells [51]. These bioactive molecules affect the physiology and behavior of recipient cells (Table 1) [52,53]. In eukaryotic hosts, EVs pack cargoes to deliver them to distant target cells. This mediates cell-to-cell communication, cell maintenance, cell proliferation, and tumor development [40]. In contrast, BEVs are used to infect the host, modulate host cell pathophysiology, evade the immune system, kill competing bacterial species, and develop antibiotic resistance [45]. OMVs and CMVs can exert these functions, but the roles of O-IMVs in interkingdom signaling are still unknown [46].

### 2.2. Roles of EV in GBA 

#### 2.2.1. Microbial EVs

**Figure 3:**
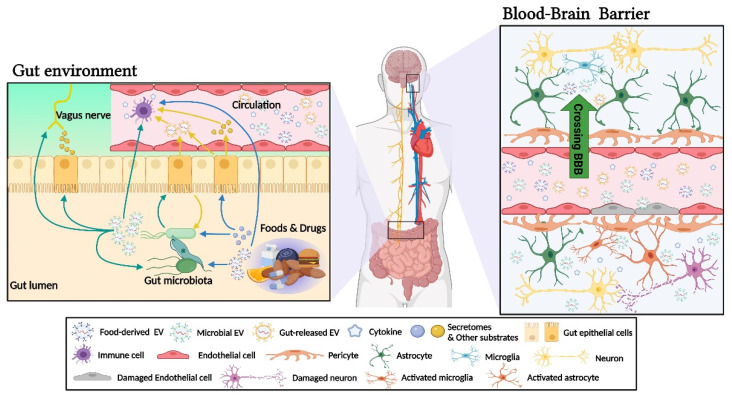
**Gut–brain interaction mediated by EVs in the gut microenvironment.** EVs occurring in the gut microenvironment can be categorized into two origins, which are microbial origin and gut cell-released origin. In addition, EVs from foods can also be considered in this environment. All the categories can enter the blood circulation across the gut epithelial barrier. Hence, they can reach and affect the immune system and other organs by traveling through the circulation, which can further affect the BBB and brain. The mechanism of vagal stimulation mediated by EVs in the gut environment is still unclear. There are several studies suggesting that bacterial EVs may interact with the vagus nerve. It can also be postulated that microbial EVs stimulate gut cells such as enteroendocrine cells to indirectly exert an influence on the vagal pathway via gut products such as gut hormones. This illustration was created on BioRender.com.

Several studies imply that bacterial OMVs can cross the gut epithelium and enter the bloodstream [54]. In addition, recent research has revealed that BEVs can efficiently cross the blood-brain barrier (BBB) and deliver cargo (Figure 3) [44]. In particular, lipopolysaccharide (LPS) is present in OMVs, which likely contributes to neuroinflammation [55]. Furthermore, OMVs can disrupt BBB integrity, which may help other harmful substrates and other BEVs across the BBB, resulting in impacts on brain pathology [44,56]. Once they enter the brain, BEVs can directly influence neurological function and trigger pathological pathways. For example, OMVs purified from the gut bacteria of AD patients presented increased BBB permeability, increased tau phosphorylation via GSK-3β, activated microglia and astrocytes, and induced neuroinflammation when injected into mice [57]. A few studies have recently revealed that BEVs can interact with the vagus nerve (Figure 3) [14,58]. Lee et al. pointed out that *Paenalcaligenes hominis*, particularly its EVs, can cause cognitive-function-impaired disorders such as AD. These EVs may penetrate the brain through the blood as well as the vagus nerve [59].

EVs released by protozoans can also be taken up by mammalian cells, and the presence of these EVs potentially modulates the entire process of host pathogenesis [35]. It has been observed that EVs from pathogenic free-living amoeba, *Acanthamoeba castellanii*, destabilize epithelial and BBB cells and induce necrotic or apoptotic cell death [60]. In addition, EVs from protozoans can modulate host immunity, gut microbiome, and gut physiology [61,62], which may affect the brain indirectly. In addition to bacteria and protozoans, the human gut can harbor many other microorganisms, such as fungi, whose EVs can also interact with host cells and participate in pathogenesis [37,38,39].

#### 2.2.2. Gut-Released EVs

The properties of EVs are affected by not only the type of parent cells but also the current physiological conditions of these cells. Enterocytes in the gut lumen are always exposed to different agents, including food, gut microorganisms, and pathogens. Changes in this dynamic environment can alter the production and properties of gut-released EVs, which may influence brain pathophysiology (Figure 3).

Irritants, drugs, or components in foods can alter the production and properties of gut-released EVs [63,64,65]. These EVs may cross the BBB and influence the physiology and behavior of the brain. Katakura et al. treated human gut epithelial Caco-2 cells with γ-aminobutyric acid (GABA) and found that GABA-induced gut exosomes facilitated neurite growth in SH-SY5Y cells [66]. They also confirmed that exosomes from the serum of oral-GABA-administered mice promoted neurite growth [67]. The direct effect of dietary GABA on sleep and stress in humans is still controversial because it has long been considered that GABA cannot cross the human BBB [68,69]. Katakura’s studies indicate that the beneficial effects of dietary GABA on the brain may be mediated by gut-released EVs.

Gut-released EVs can also be influenced by microbial infection, toxins, and even EVs from microorganisms [70,71,72]. This microorganism–host interaction can have an effect on other remote organs [73]. Adherent-invasive *Escherichia coli* (AIEC), such as *E. coli* LF82, a major contributor of Crohn’s disease (CD), is known to use OMVs to facilitate the invasion of human gut cells [74,75]. LF82-infected gut cells produce larger exosomes [76] and upregulate certain micro RNAs (miRNAs) in their exosomes, such as miR-130a [77]. Some studies have reported that miR-130a increases BBB permeability and reduces occludin expression in the BBB [78,79]. In addition, various abnormalities in the brain have been observed in CD patients, such as an increased incidence of psychological stress, mood disorder, and neurological sequelae [80]. It is suggested that these abnormalities in CD patients may be due to EVs from infected gut cells.

In addition to xenosubstrates and microorganism–host interactions, other issues in the gut, such as hemorrhagic shock, may lead to changes in the properties of gut-released EVs [81]. Chen et al. have confirmed that microglia incorporate gut ischemia/reperfusion-induced gut exosomes both in vivo and in vitro and increase neuronal apoptotic rates and decrease synaptic stability in mice [82].

Many studies have reported that gut-released EVs influence the immune system [81]. Exosomes secreted by gut epithelial cells bear exogenous peptides complexed to major histocompatibility complex class II (MHC-II), and they preferentially interact with dendritic cells, resulting in greatly enhanced peptide presentation to T-cells [83]. This interaction activates the gut immune system. The activation of the immune system in the gut occurs in response to neuroinflammation and brain injury as well as changes in neurogenesis and plasticity [84]. In addition, due to an increasing body of evidence, the existence of more complex communication systems such as the gut–liver–brain axis or the gut–kidney–brain axis can be speculated [85,86], and EVs might also be involved in these systems. For example, gut-dysbiosis-derived exosomes can trigger hepatic steatosis by directly affecting the liver in mice [73]. As both gut dysbiosis and hepatic steatosis are related to brain-associated disorders [87,88], gut–liver–brain interactions could be mediated by gut-released EVs.

### 2.3. EVs from External Source 

In addition to microbial or gut-released EVs, EVs originating from external sources, such as foods, can cross the gut epithelium and enter the blood circulation [89,90]. For instance, human macrophages can take up food-derived EVs (FDEs) from dietary bovine milk [91]. Matic et al. reported that FDEs from dietary bovine milk also reduced apoptosis and drug-induced cytotoxicity of murine RAW 264.7 macrophages [92]. Furthermore, FDEs have the potential for alleviating diseases and modulating gut microbiota [93]. Teng et al. demonstrated that ginger’s FDEs could modulate the composition and localization of gut microbiota, which eventually affected host physiology [94]. Therefore, there are possibly many routes where FDEs affect brain physiology and behavior.

The direct impact of FDEs on the brain has been demonstrated by recent studies. Zempleni et al. demonstrated that orally administered dietary milk FDEs were distributed in various organs, including the brain, in mice [95]. Based on this observation, they conducted a cognitive test using C57BL/6 mice after oral administration of these FDEs [96,97]. They concluded that depletion of dietary milk FDEs could affect the cognitive ability of mice.

## 3. GBA-on-a-Chip for Research on EVs in Gut Microenvironment

### 3.1. Multi-Organ-on-a-Chip as a Novel In Vitro Platform for GBA Research

**Figure 4:**
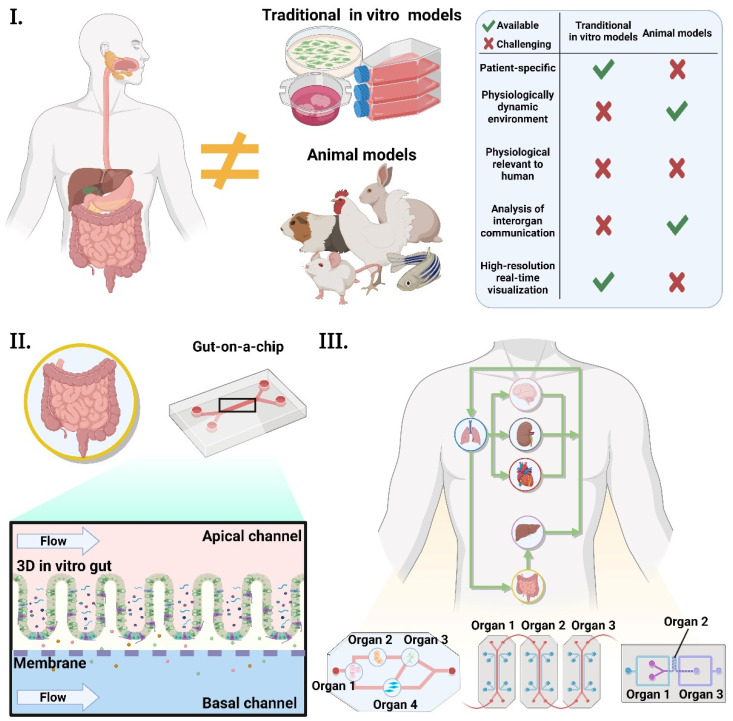
**Concept of organ-on-a-chip.** (**I.**) Conventional in vitro models and animal models are physiologically different from the human body. They also hinder the understanding of human diseases and the development of new therapeutic strategies. (**II.**) The concept of organ-on-a-chip is to mimic the dynamic 3D microenvironment of an organ at a small scale. In the example of a single-organ gut model, the gut cells form a 3D villi structure, and this gut epithelium is exposed to constant fluidic shear. This villi structure helps the cultured cells to feature more physiologically relevant characteristics. The fluidic flow gives the cells the physiologically relevant mechanical stimulation seen in the human gut and washes out the wastes from the cells. (**III.**) Multi-organs-on-a-chip incorporates multiple organs in a single platform. These organs are interconnected through microchannels, which allow cross-organ communication. Generally, these multi-organ models are more physiologically relevant than single-organ models since diseases or the effects of drugs are developed and progressed through multi-organ interactions. These illustrations were created on BioRender.com.

Animal models have been mainly used in GBA research [18]. However, they require experienced handling [98] and show poor experimental reproducibility [19], while real-time analysis of their responses is challenging [99]. Furthermore, extrapolation of animal data to humans can be problematic, and detailed studies into the underlying mechanisms can often be difficult due to physiological discrepancies between mammalian species [13,100]. Meanwhile, conventional in vitro models, such as multi-well plates, feature a well-established methodology and are easy to handle and cost-effective, having been widely used in the research field [20]. However, they fail to reproduce the dynamic tissue environment, such as fluid flow, blood vessels, and cell-cell interactions [20]. For these reasons, drug discovery and health research using these models have been quite problematic (Figure 4). Therefore, novel effective models and approaches are required.

OOCs have unique advantages; they can capture physiological processes that cannot be reproduced with conventional in vitro models such as multi-well plates [101]. They can mimic the physiological environment of an organ, with the ability to regulate key parameters, including concentration gradients, shear force, cell patterning, tissue boundaries, and tissue–organ interactions (Figure 4) [102]. Furthermore, various physical, chemical, and biological sensors can be incorporated into OOCs. These sensors have been shown to provide reproducible results, with data transmission, multiplexing, and online monitoring capability, which allows for improved time resolution, continuous measurements, and a rapid read-out [103,104].

Multi-organs-on-a-chip (MOOC) cultures cells of different organs and tissues simultaneously, which are connected by microchannels to achieve multi-organ integration (Figure 4) [102,105]. These models can potentially mimic the human physiology of multiple organs interacting with one another [106]. MOOCs have been used to observe continuous or linked pharmacokinetic processes such as absorption, distribution, metabolism, and excretion (ADME) of diverse drug administration pathways [26,107]. The obtained data can be used to construct mathematical models (in silico models) for the prediction of drug efficacy in the body [26,107,108,109,110].

In addition, MOOCs can be used as disease models since many diseases are developed and progressed via cross-organ communication. Currently, single-organ models using OOCs have been well-established, but they cannot recapitulate the inter-organ communication seen in the in vivo human body. For example, inter-organ communication is important in the context of cancer metastasis [111]. Multi-organ models that combine tumor and potential metastatic niches can elucidate the processes involved in the metastatic cascade and result in possible new treatments [111]. A good example for utilizing MOOCs to study metastasis processes, especially in the brain, is the research conducted by Liu et al. in 2019 [112]. They discovered that AKR1B10 (aldo-ketoreductase family 1 B10) promoted the brain metastasis of lung cancer cells using their lung–brain axis-on-a-chip. This kind of brain disease model, using MOOCs, could provide a blueprint for GBAs-on-a-chip.

### 3.2. Considerations on the Design of GBA-on-a-Chip

#### 3.2.1. BBB-on-a-Chip

To the best of our knowledge, very few GBAs-on-a-chip have been reported, and we currently lack a realistic platform to investigate the inter-organ communication between gut and brain [113]. Raimondi et al. provided a blueprint of a GBA-on-a-chip through their review paper [18]. There are two major biological barriers in the GBA: the gut barrier and the BBB [114]. The integrity of these barriers is known to be closely associated with the pathology and behavior of the brain [115]. This is because the gut epithelial barrier keeps harmful xenobiotic compounds from entering systemic circulation, and the BBB plays a vital role in maintaining the physical and chemical homeostasis of the brain and protects the brain from harmful molecules and pathogens in the blood [116,117,118]. Due to this physiological significance, the incorporation and maintenance of these barriers are essential for constructing in vitro GBA models.

**Figure 5:**
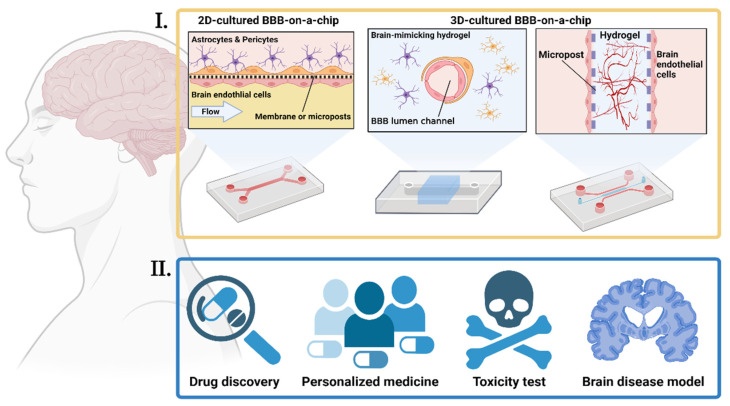
**Various BBBs-on-a-chip and their application.** (**I.**) Strategies for reconstructing the in vitro BBB in an OOC platform are diverse. Cells can be cultured in a 2D environment under fluidic flow or 3D-cultured with different approaches, such as seeding in hollow hydrogel or the angio/vasculogenesis approach. (**II.**) The BBB-on-a-chip can be applied to drug development, patient-specific medicines, toxicological study, and research on brain diseases. This illustration was created on BioRender.com.

The BBB consists of three types of cells: brain endothelial cells (BECs), astrocytes, and pericytes [119]. Communication between these cells is indispensable for the maintenance of brain homeostasis. Pericytes stabilize blood vessels, and astrocytes help maintain barrier properties. The lumen of the BBB formed by BECs is always exposed to the fluidic shear force generated by blood circulation [119]. Studies have shown that shear stress improves in vitro BBB integrity [119,120]. Therefore, the presence of shear stress is necessary for constructing an in vitro BBB. In addition to mechanical stimulation, structural considerations are necessary for the in vitro BBB. In a 2D-cultured BBB-on-a-chip, accurate representations of the BBB microenvironment are often limited by microfabrication (i.e., photolithography) and commercial microporous membranes [121]. For example, interactions of BECs, astrocytes, and pericytes are blocked by microposts or membranes in the 2D-cultured BBB-on-a-chip. Recently, 3D-cultured BBB models using novel approaches have been suggested. For instance, BECs have been seeded in cylinder microtubes in hydrogel, and the angio/vasculogenesis of BECs has been induced in microfluidic chips (Figure 5) [119].

In recent years, researchers have established brain disease models using BBBs-on-a-chip (Figure 5). One of the great examples was a study conducted by Shin et al. [122]. They reproduced AD conditions by co-culturing neuronal cells and BECs in a chip whose channels were arranged in parallel in a single layer. This model recapitulated several key aspects of BBB dysfunction observed in patients with AD. Vatine et al. reconstructed a patient-specific BBB model using induced pluripotent stem cells (iPSCs) from several patients [123]. They could recapitulate the general functions and inflammatory responses of the BBB, but, more importantly, the iPSC-based BBB-on-a-chip could detect functional differences in the BBB in patients.

#### 3.2.2. Gut-on-a-Chip

It is necessary to consider the environment, structure, and function of the gut to design a realistic in vitro gut model [124,125]. Generally, traditional 2D cell culture is physiologically different from the in vivo gut epithelium, which features a 3D architecture [126]. Culturing cells in 3D space offers cells more physiologically realistic conditions and allows the cells to present more authentic functions [126]. Yu et al. reported that gut epithelial cells cultured on a 3D hydrogel villi scaffold featured both absorption and differentiation more similar to in vivo conditions than those achieved by 2D culture [127]. We also report that gut epithelial cells cultured on 3D collagen villi showed improved production of MUC17, which is one of the mucins required to protect the gut epithelium against pathogenic bacterial infection [128]. Furthermore, this collagen villi can stimulate CYP3A4 and alkaline phosphatase activities of the gut epithelial cells; these function as metabolic enzymes and gut epithelial differentiation markers, respectively [129]. Meanwhile, we attempted to incorporate 3D hydrogel villi into a gut-on-a-chip [130]. We have demonstrated that the combination of fluidic stimulus and 3D structure can induce further improvement in gut function [131]. In our study, the 3D villi structure with fluidic stimulation facilitated enzyme activity and altered the absorption profile of the epithelia to different drugs.

#### 3.2.3. GBA-on-a-Chip

Simulating the diverse organ-specific microenvironment of different organs in a single MOOC platform can be extremely challenging. To address this problem, a modular approach has been conceived. Modular MOOCs consist of multiple organ modules [111,132]. Modularized MOOCs provide convenience for analyzing and customizing individual organ modules [133,134]. We showed that the modular approach can offer individualized environments for each cell type by culturing them separately before co-culturing [134]. Many of the current modular MOOCs feature a main body platform and multiple organ modules that can be inserted into the main body, and this main body can recapitulate in vivo-like circulation routes, which allow interconnection between the different organ modules [133,135,136]. Recently, Trapecar et al. established a modular MOOC to simulate gut–liver–brain interactions in the context of PD [135]. In their platform, they incorporated a main body circulation system to connect each module in the gut–liver–brain axis-like circulation route. Using this platform, they successfully mimicked in vivo-like behavior in cerebral modules and found that microbiome-associated short-chain fatty acids increased the expression of pathology-associated pathways in PD patients. However, this model has several limitations. First, this platform lacked direct exposure of the gut module to shear force, which can influence the physiological responses of the in vitro gut. Second, the researchers did not incorporate the BBB in their model, which is another essential barrier in the context of GBA.

### 3.3. GBA-on-a-Chip for Models of EV-Mediated Diseases

#### 3.3.1. Investigating Organ Level Response Using Isolated EVs

Applying EVs directly into target cells is a straightforward method for investigating the cellular response to these EVs. However, the treatment in traditional 2D culture systems can be problematic because it is quite challenging to analyze intercell communication and mimic physiologically relevant environments [137]. OOCs enable the control of local concentrations of molecular signals such as growth factors, chemokines, and hormones, allowing the study of the interactions between EVs and organ-specific cells [138]. Moran et al. isolated endothelial cell-derived EVs and treated their human-heart-on-chip with them [139]. Their experiment showed that endothelial EVs could rescue ischemia-reperfusion injury. This approach showed that observing organ-specific interactions with EVs is possible. However, the isolation of EVs is usually a time-consuming and labor-intensive practice [21,22,23,24]. In 2017, Woo et al. reported a lab-on-a-disc platform for rapid, label-free, and highly sensitive EV isolation, called Exodisc [140]. It requires only 30 min and a low gravitational force of 500 g to isolate EVs, whereas traditional ultracentrifugation requires 100,000 g and 11 h for isolation. This Exodisc-assisted approach was demonstrated by Kim et al. to study EV-mediated breast cancer metastasis in the liver using a liver-on-a-chip model [141]. With the rapid EV isolation method, OOCs can be effectively used to study EV-mediated diseases.

#### 3.3.2. Real-Time Tracking of Cell-to-Cell EV Delivery in an Organ-on-a-Chip

Due to the complex nature and very small size of EVs, it may be difficult to elucidate their specific roles in various signaling pathways [142]. Visualizing and tracking EVs in real-time is crucial to understanding the biological mechanisms of the potential of EVs [143]. For example, Morad et al. found that breast-cancer-derived EVs breached an intact BBB and identified transcytosis as the mechanism underlying this process using fluorescent protein-labeled EVs with a combination of in vivo and in vitro models, including a BBB-on-a-chip [144].

Surface labeling methods, such as the lipophilic dye method, have been commonly used in OOC-based EV research [138]. It is suitable for observing single-organ-level interaction but not desirable for studying cross-organ interaction via live cell-producing EVs. In this case, making live cells produce labeled EVs is a promising strategy. Oh et al. transfected cells with a cytomegalovirus-driven GFP-tagged CD63 vector to make cells secrete GFP-labeled exosomes [145]. They could track single exosomes in real-time with their designed cell-culturing microfluidic chip during cell-to-cell exosome delivery. However, their study required extensive additional experiments to demonstrate that the specific exosomal cargo of interest contributed to cell differentiation since this method could not mark the inner cargoes of exosomes.

In a subsequent study, they represented direct and real-time tracking of single exosomes that contained the specific miRNA of interest during cell-to-cell exosome delivery using a graphene-oxide quenching-based molecular beacon imaging technique [146]. This technique could label specific exosomal miRNA inside of cells before being released through exosomes. Additionally, their designed microfluidic device made real-time target exosomal miRNA tracking more feasible, and they could demonstrate that the specific miRNA of interest was involved in cell differentiation. This method could be applied to determine which specific exosomal miRNAs in the gut affect brain pathophysiology.

#### 3.3.3. Studying EV Distribution and Tropism Using Multi-Organ-on-a-Chip

The type of parent cell can influence the organ-specific tropism of EVs. For instance, EVs from dendritic cells show increased accumulation in the spleen, whereas melanoma EVs are more likely to accumulate in the lungs [147,148]. Because of this property, the drug efficiency or pathology determined by EVs can be affected, which is why the distribution of pathogenic or drug carrier EVs in the body must be investigated. Animal models are not suitable for studying EV distribution in the body because there is a physical discrepancy between mammalian species and these models are limited to low resolution when it comes to real-time EV tracking [137]. MOOCs may be promising models for this purpose. Tian et al. demonstrated that breast-cancer-derived EVs had stronger liver tropism than kidney using their liver–kidney-on-a-chip [149]. Similar results were observed in the animal experiments conducted by the same authors. In addition, they compared the Transwell model with the chip and presented that the fluid flow in the chip facilitated EV uptake. Several recent studies have reported similar observations. Papademetriou et al. demonstrated that the transport of ligand-attached liposomes across the BBB was facilitated in the presence of fluidic flow [150]. More recently, we also confirmed that the presence of fluidic flow facilitated the transport of gut-released EVs across the gut epithelium to the BBB [13]. Consequently, MOOCs may be a great platform for studying EV distribution and tropism in the body. This approach could be highly useful for studying the potential of EVs as drug carriers or therapeutic agents targeting the brain. 

**Figure 6:**
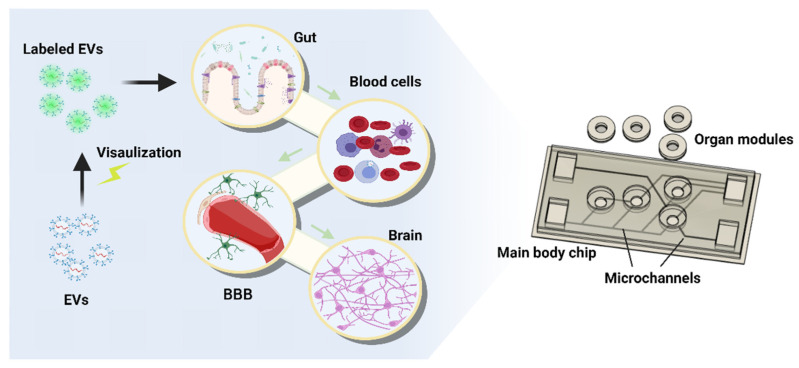
**Modular MOOC-based GBA model for studying EV-mediated gut–brain interaction.** The modular MOOC-based GBA-on-a-chip contains several organ modules that will be inserted into the main body platform. The main body features an in-vivo-like circulation route that connects each element of the GBA. The modular approach provides convenience for analysis and allows customized experiment protocols by replacing the modules with those with special treatment. EVs present organ-specific tropism, which is why it is vital to study their distribution in the body. OOCs, especially modular MOOCs, can be a suitable candidate to conduct this concept of research. This approach can get more feasible when incorporating labeled EVs for observing real-time delivery since real-time changes can be captured in many OOC platforms. This illustration was created on BioRender.com.

Highly modularized GBAs-on-a-chip could be an ideal platform for studying the interaction of gut-derived EVs with various organs in the body (Figure 6). Analyzing EVs in multi-organ GBA-on-a-chip models should be relatively easy as fluids in the platform can be collected for analysis [111]. The visualization or labeling of EVs for real-time analysis of the transport and behavior of EVs during cell–cell or organ–organ interaction could potentially reveal valuable information about the mechanism of action of EVs in the physiological context. 

## 4. Limitation

OOCs have great potential for studying EV-mediated diseases and testing EV therapeutics, yet more complex platforms are generally more difficult and expensive to make [137]. The size of the organ, the flow and shear in each organ module, and the total volume of medium must be considered to achieve physiological relevance while meeting physical limitations [111]. However, it is still challenging to precisely replicate the physical and chemical microenvironment of in vivo tissues as well as the dynamics of organ interactions in MOOC [106]. To deal with this issue, introducing additional engineering concepts or applying mathematical/computer models will be helpful [106].

Additionally, the visualization of EVs is vital to understanding the mechanism of EV-mediated diseases. Nevertheless, there are limitations since the inner content of EVs needs to be marked for visualization. To uncover the roles of EV cargoes, efficient isolation of EVs may be indispensable for further analysis. Recently, novel microfluidic systems and sensing technologies for EV isolation and analysis have been suggested. These microfluidic devices can efficiently isolate EVs from small volumes of biofluid [151], and new sensing technologies can analyze inner contents without bulk isolation [152]. Incorporating these technologies with OOC platforms may be necessary for EV research using OOCs.

## 5. Conclusions

To date, research on the EV-mediated GBA has focused on the roles of BEVs in the gut microenvironment. However, various other types of EVs, such as gut-released EVs, should also be considered. Recent studies suggest the involvement of gut-released EVs in gut–brain interaction. Elucidating the role of EVs in GBA communication is essential for understanding the mechanism of many important diseases.

There are many critical challenges in current approaches for studies on GBA and EVs, which can hinder the progress of EV-mediated GBA research. We speculate that recent advances in OOC technology could provide a promising solution. In this review, we provided a summary of recent relevant studies as well as strategies of applying GBAs-on-a-chip for studying the role of EVs in the GBA. In conclusion, we believe that modularized GBAs-on-a-chip could provide a useful platform for real-time analysis of EVs in various pathophysiological contexts such as the gut–liver–brain axis or the gut–kidney–brain axis (Figure 6).

## Figures and Tables

**Table 1:** Characteristics of different extracellular vesicles.

Origin	Eukaryotes			Gram-Negative Bacteria		Gram-Positive Bacteria
Type	Exosome	MV	AB	OMV	O-IMV	CMV
Surface compounds	CD63, TSG101, alix, flotillin	Integrin, selectin, flotillin-2	Thrombospondin, C3b	LPS	LPS	LTA
Contents	Protein, lipid, RNA and DNA	Protein, lipid, RNA, and DNA	Cytosolic content (protein, RNAs, fragmented DNA) and cellular organelles	DNA, RNA, periplasmic proteins, peptidoglycans, enzymes, and toxins	DNA, RNA, ATP, periplasmic proteins, cytoplasmic proteins, phages, endolysin, and toxins	DNA, RNA, cytoplasmic proteins, phages, endolysin, enzymes, and toxins
Biological purposes	Packing cargoes to deliver them to distant cells, involving cell–cell communication, cell maintenance, cell proliferation, and tumor progression	Similar to exosomes	Little known	Bacteria adhesion/invasion, modulation of host cell pathophysiology, host immune evasion, killing competing bacterial species, and antibiotics resistance	Role in interdomain signaling is unclear	Bacteria adhesion/invasion, modulation of host cell pathophysiology, killing competing bacterial species, and antibiotics resistance
References	[40,41,42]	[40,42]	[40,41,43]	[17,33,44,45]	[30,33,46]	[31,32,33,45,47,48]

MV, microvesicle; AB, apoptotic body; OMV, outer membrane vesicle; O-IMV, outer-inner membrane vesicle; CMV, cytoplasmic membrane vesicle; LPS, lipopolysaccharide; LTA, lipoteichoic acid.

## Data Availability

Not applicable.
